# Parapapillary Atrophy: Histological Gamma Zone and Delta Zone

**DOI:** 10.1371/journal.pone.0047237

**Published:** 2012-10-18

**Authors:** Jost B. Jonas, Shefali B. Jonas, Rahul A. Jonas, Leonhard Holbach, Yi Dai, Xinghuai Sun, Songhomitra Panda-Jonas

**Affiliations:** 1 Department of Ophthalmology, Medical Faculty Mannheim of the Ruprecht-Karls-University Heidelberg, Germany; 2 Department of Ophthalmology, Friedrich-Alexander University Erlangen-Nürnberg, Erlangen, Germany; 3 Department of Ophthalmology and Vision Science, Eye and ENT Hospital, Shanghai Medical College, Fudan University, Shanghai, China; 4 State Key Laboratory of Medical Neurobiology, Institutes of Brain Science, Fudan University, Shanghai, China; University of Sydney, Australia

## Abstract

**Background:**

To examine histomorphometrically the parapapillary region in human eyes.

**Methodology/Principal Findings:**

The histomorphometric study included 65 human globes (axial length:21–37 mm). On anterior-posterior histological sections, we measured the distance Bruch's membrane end (BME)-optic nerve margin (“Gamma zone”), BME-retinal pigment epithelium (RPE) (“Beta zone”), BME-beginning of non-occluded choriocapillaris, and BME-beginning of photoreceptor layer. “Delta zone” was defined as part of gamma zone in which blood vessels of at least 50 µm diameter were not present over a length of >300 µm. Beta zone (mean length:0.35±0.52 mm) was significantly (P = 0.01) larger in the glaucoma group than in the non-glaucomatous group. It was not significantly (P = 0.28) associated with axial length. Beta zone was significantly (P = 0.004) larger than the region with occluded choriocapillaris. Gamma zone (mean length:0.63±1.25 mm) was associated with axial length (*P*<0.001;r^2^ = 0.73) with an increase starting at an axial length of 26.5 mm. It was not significantly (*P* = 0.24) associated with glaucomatous optic neuropathy. Delta zone (present only in eyes with axial length of ≥27 mm) was associated with axial length (*P* = 0.001) and scleral flange length (*P*<0.001) but not with glaucoma (*P* = 0.73).

**Conclusions/Significance:**

Parapapillary gamma zone (peripapillary sclera without overlying choroid, Bruch's membrane and deep retinal layers) was related with axial globe elongation and was independent of glaucoma. Delta zone (no blood vessels >50 µm diameter within gamma zone) was present only in highly axially elongated globes and was not related with glaucoma. Beta zone (Bruch's membrane without RPE) was correlated with glaucoma but not with globe elongation. Since the region with occluded choriocapillaris was smaller than beta zone, complete loss of RPE may have occurred before complete choriocapillaris closure.

## Introduction

Previous clinical and histological studies have shown that the parapapillary region of normal eyes, myopic eyes and eyes with glaucomatous optic neuropathy show distinct features, which have so far been summarized into an alpha zone and a beta zone [1]. Alpha zone was defined as irregular hyperpigmentation and hypopigmentation and it was located in the periphery of the parapapillary atrophy. Beta zone was characterized by visible sclera and visible large choroidal vessels and a location between the peripapillary scleral ring and alpha zone. Correspondingly, histological studies in human eyes and monkey globes confirmed in clinical histological correlations that alpha zone corresponded histologically to irregularities in the retinal pigment epithelium (RPE), and that beta zone showed a complete loss of RPE cells and an almost complete loss of photoreceptors and a closure of the choriocapillaris [2]–[5]. This scheme of only two zones in the parapapillary region has recently been challenged. Recent histological investigations revealed, that the parapapillary region in highly myopic eyes showed an additional feature: highly myopic eyes demonstrated a markedly elongated and thinned peripapillary scleral flange and a widening of the peripapillary orbital cerebrospinal fluid space [4]. This finding was corroborated by clinical studies [6]. For medium myopic eyes, recent clinical investigations using optical coherence tomography have shown another feature: between a beta zone (characterized by Bruch's membrane) and the border of the optic disc, the images demonstrated a zone which was composed simply of sclera and overlying retinal nerve fiber layer tissue [7]–[14]. We therefore, re-assessed histological slides of human globes without and with axial elongation and without and with glaucoma.

## Methods

### Ethics Statement

The ethics commission II of the Medical Faculty Mannheim of the Ruprecht-Karls-University Heidelberg, Germany, approved the study including that informed consent of the patients treated up to more than 30 years prior to the study was not necessary.

The study included human globes which either had been enucleated due to painful absolute glaucoma or which had been enucleated because of a malignant choroidal melanoma. In the glaucomatous group, vision was completely or almost completely lost, and enucleation became necessary usually due to intractable pain which could not be treated by medication. The information from the clinical charts was not sufficient to reliably describe the duration of glaucoma, the height of intraocular pressure, and what types of medications had been used. Since pain was the usual reason for enucleation in the glaucoma group and since phthitic eyes or eyes with corneal perforations were excluded from the study, it is likely that most or all glaucoma eyes had high intraocular pressure. In the tumor group, the malignant choroidal melanomas did not infiltrate the trabecular meshwork, neither directly or indirectly by migrating cells. The parapapillary region was free of tumor cells. At the time when the eyes were enucleated, no other treatment modalities such as radiologic brachytherapy were available or were thought not to be suitable for tumor removal with respect to its location and size. The globes had already been included in previous histomorphometric studies [4]. Immediately after enucleation, the globes were fixed in a solution of 4% formaldehyde and 1% glutaraldehyde and processed for histological sectioning. In a routine histo-pathologic work-up, the axial length of the globes was measured after the fixation and before sectioning of the eyes. A ruler was used to determine the axial length measurements. The measurements were recorded in one millimeter steps. The depth of the anterior chamber was not measured due to fixation related changes in the thickness of the lens and its position. The globes were prepared in a routine manner for light microscopy. An anterior-posterior segment going through the pupil and the optic nerve was cut out of the fixed globes. These segments were dehydrated in alcohol, imbedded in paraffin, sectioned for light microscopy, and stained by the Periodic-Acid-Shiff (PAS) method or by hematoxylin-eosin. For all eyes, one section running through the central part of the optic disc was selected for further evaluation.

**Figure 1:**
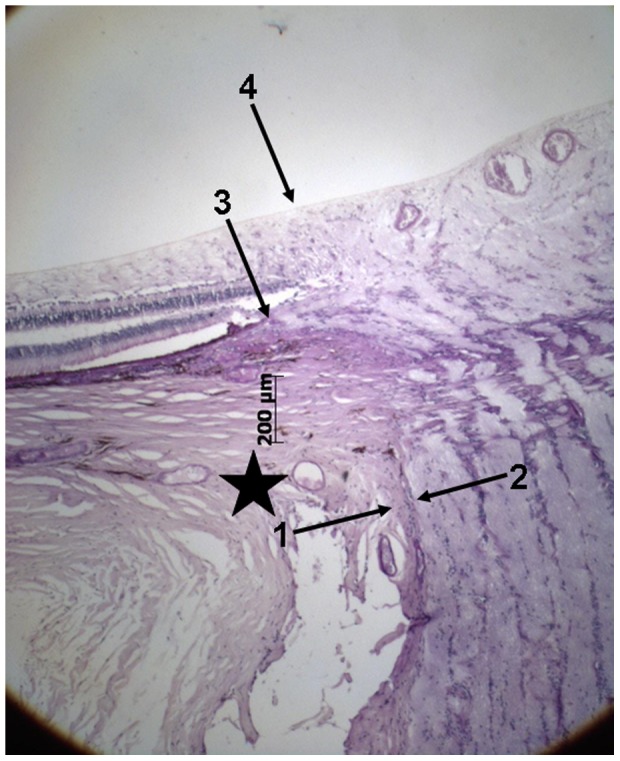
Photomicrograph of the optic nerve head (staining: PAS); arrows #1 and #2: pia mater of the optic nerve; arrow #3: end of Bruch's membrane; arrow #4: projection of the outer margin of the pia mater of the optic nerve; Gamma zone: region between arrows #3 and #4; black star: beginning of dura mater of the optic nerve.

**Figure 2:**
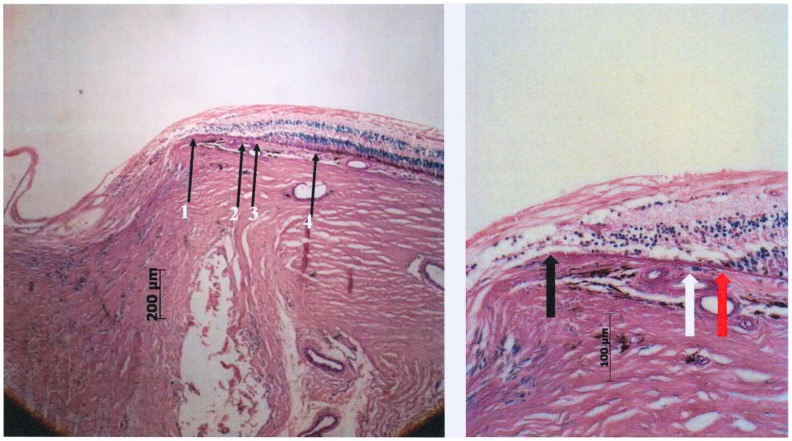
Photomicrograph of a glaucomatous optic nerve head (staining: PAS). Left: arrow #1 end of Bruch's membrane; arrow #2: beginning of choriocapillaris not occluded beneath Bruch's membrane; arrow #3: beginning retinal photoreceptors on Bruch's membrane; arrow #4: beginning of retinal pigment epithelium cells on Bruch's membrane. Right: higher magnification of beta zone; Black arrow: End of Bruch's membrane at the optic disc border; White arrow: open choriocapillaris; Red arrow: end of photoreceptor layer, choriocapillaris open.

At the posterior pole of the globes, we assessed (Fig. 1,2)

**Figure 3:**
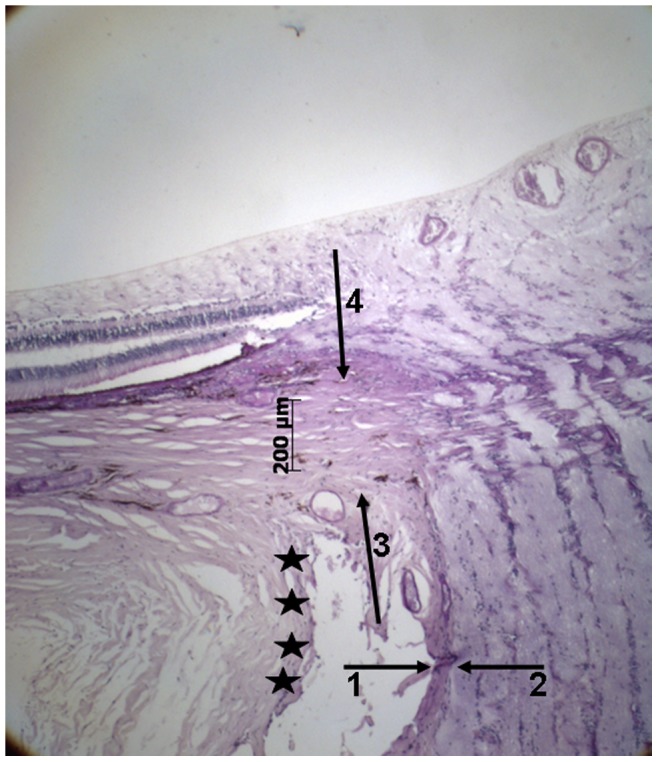
Photomicrograph of the optic nerve head (staining: PAS); arrows #1 and #2: pia mater of the optic nerve; arrows #3 and #4: peripapillary scleral flange as roof of the orbital cerebrospinal fluid space; black stars: dura mater of the optic nerve.

— the distance between the end of Bruch's membrane and the outer margin of the optic nerve (covered by pia mater) (“Gamma zone”) (Fig. 1)
— the distance between the end of Bruch's membrane and the dura mater of the optic nerve meninges (Fig. 1)
— the distance between the end of Bruch's membrane and the beginning of RPE (“Beta zone”) (Fig. 2a, b)
— the distance between the end of Bruch's membrane and the beginning of the choriocapillaris (not occluded) (Fig. 2a, b)
— the distance between the end of Bruch's membrane and the beginning of retinal photoreceptors (Fig. 2a, b)
— the length and thickness of the scleral flange (Fig. 3)

**Figure 4:**
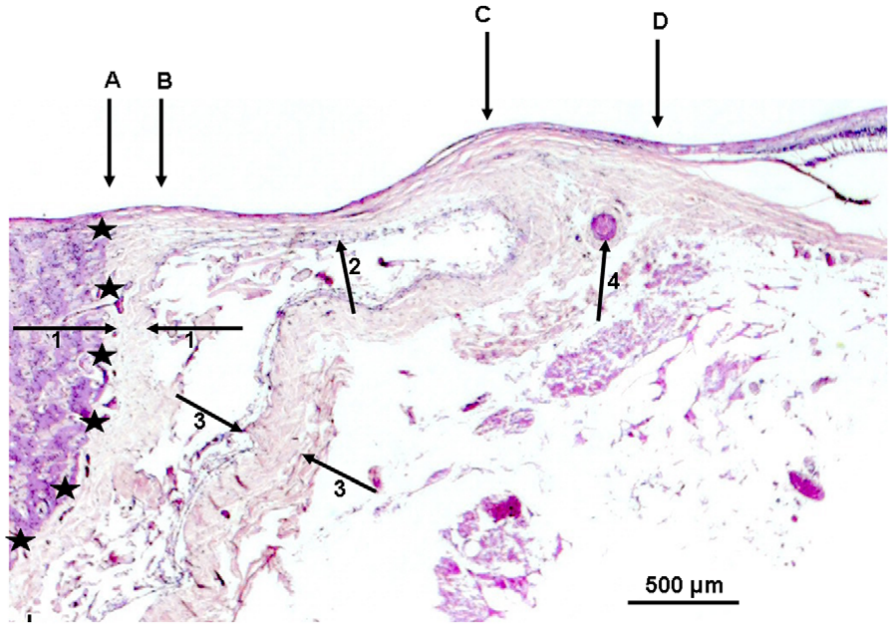
Photomicrograph of a highly myopic optic nerve head (staining: PAS); black stars: inner border of the optic nerve; arrows #1: pia mater of the optic nerve; arrows #2: peripapillary scleral flange as roof of the widened orbital cerebrospinal fluid space; arrows #3: dura mater of the optic nerve; arrow #4: arterial circle of Zinn-Haller; A–B: Pia mater (potentially the “peripapillary scleral ring” upon ophthalmoscopy); B–C: Delta zone (no blood vessels >50 µm diameter within gamma zone); C–D: remaining Gamma zone (peripapillary sclera without overlying choroid, Bruch's membrane and deep retinal layers).

Additionally we measured the thickness of the sclera at the limbus, ora serrata, equator, midpoint between the posterior pole and the equator, posterior pole and outside of the optic nerve head after merging of the optic nerve sheaths with the sclera. As “gamma zone”, we defined the region between the outer margin of the optic nerve (covered by pia mater) and the end of Bruch's membrane, if the end of Bruch's membrane did not overhang into the region of the optic nerve head (Fig. 1). “Delta zone” was a central part of gamma zone in which blood vessels of at least 50 µm diameter were not detected and which had a minimal length of 300 µm (Fig. 4). The choriocapillaris was considered not to be occluded if fine open capillaries of a diameter of about 10 to 20 µm were detectable just beneath Bruch's membrane. All measurements were performed before a discussion came up with further differentiating parapapillary atrophy into a new beta zone, a gamma zone and a delta zone. The diagnosis of glaucoma was based on the information obtained from the clinical charts and it was based on the light-microscopic appearance of the anterior chamber angle for the differentiation between open-angle glaucoma and secondary angle-closure glaucoma.

The statistical analysis was performed using a commercially available statistical software package (SPSS for Windows, version 20.0, SPSS Inc., Chicago, IL). Means and standard deviations as well as medians and ranges were presented. The distribution of the values of the parameters were tested by the Kolmogorov-Smirnov test for Gaussian distribution. Un-paired tests (Mann-Whitney-test) were used to compare beta zone length or other zones between the glaucoma and the non-glaucomatous groups. For comparing different zones within the same eyes, such as “the region of Bruch's membrane with the underlying choriocapillaris occluded'” and “beta zone” and for comparing “the region of Bruch's membrane without photoreceptors” and “beta zone'”, paired tests (Wilcoxon test) were applied. Linear regression models were used to investigate the associations between the lengths of the various regions. Variables included in the multivariate models were axial length and scleral thickness measured at various locations of the globe. Odds ratios (OR) were presented and their 95% confidence intervals (CI) were described. All *P*-values were 2-sided and considered statistically significant when less than 0.05.

## Results

The study included 65 human globes, among which 55 globes showed a glaucomatous optic neuropathy and 10 eyes had been enucleated due to a malignant melanoma of the choroid. Mean age was 63.4±11.6 years (median: 61 years; range: 45–89 years). Mean axial length was 27±3.6 mm (median: 27 mm; range: 21–37 mm). Out of the 65 globes, 34 (52%) eyes were highly elongated with an axial length >26 mm. Out of these 34 eyes, 31 (91%) eyes were glaucomatous. All measured parameters except of the axial length of the globes were not normally distributed (*P*<0.001).

Mean length of Beta zone was 0.35±0.52 mm (median: 0.14; range: 0 to 2.24 mm). In univariate analysis, beta zone was not significantly associated with age (*P* = 0.95) nor with gender (*P* = 0.16). Beta zone was significantly larger in the glaucoma group than in the non-glaucomatous group (0.32±0.54 mm versus 0.10±0.13 mm; *P* = 0.01). Beta zone size was not significantly related with vertical globe diameter (*P* = 0.70) and horizontal globe diameter (*P* = 0.15). Size of beta zone was not significantly associated with axial length (*P* = 0.28). In a similar manner, beta zone size was not significantly correlated with the thickness of the peripapillary scleral flange (*P* = 0.86) nor with scleral thickness measurements obtained at the posterior pole (*P* = 0.19), outside of the optic nerve (*P* = 0.59), at the midpoint between the posterior pole and the equator (*P* = 0.64), the equator (*P* = 0.22), the ora serrata (*P* = 0.99), and the limbus (*P* = 0.96).

The region of Bruch's membrane with the underlying choriocapillaris occluded (mean length: 0.25±0.33 mm; median: 0.14; range: 0 to 1.95 mm) was significantly (*P* = 0.004) smaller than beta zone with a mean difference of 0.11±0.33 mm.

**Figure 5:**
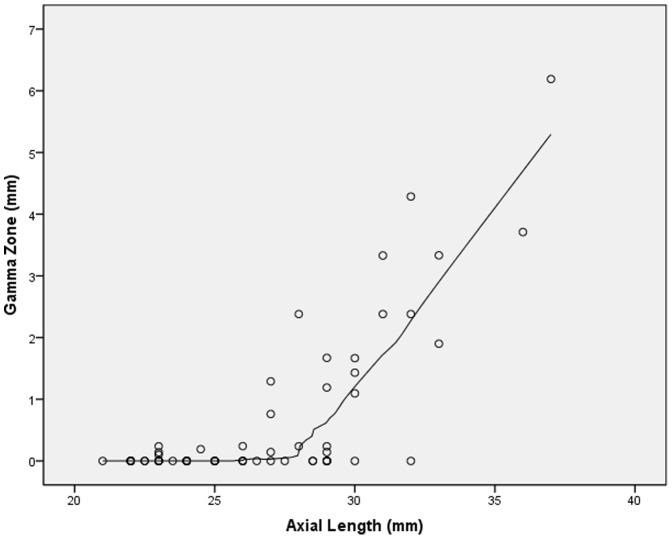
Scatterplot showing the distribution size of gamma zone versus axial length (locally weighted scatterplot smoothing (LOESS) statistics).

**Figure 6:**
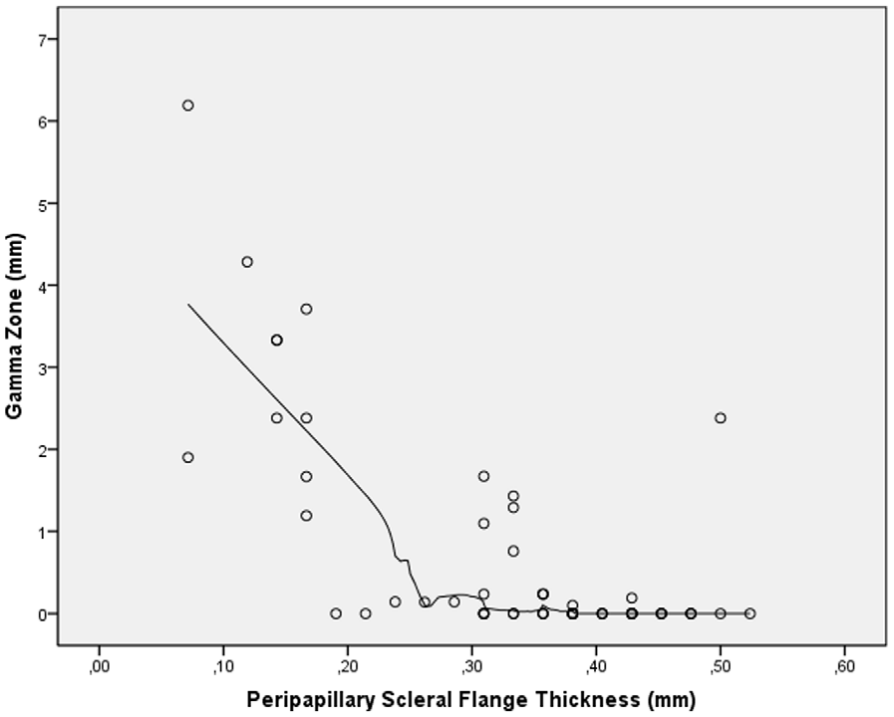
Scatterplot showing the distribution size of gamma zone versus thickness of the peripapillary scleral flange (locally weighted scatterplot smoothing (LOESS) statistics).

**Figure 7:**
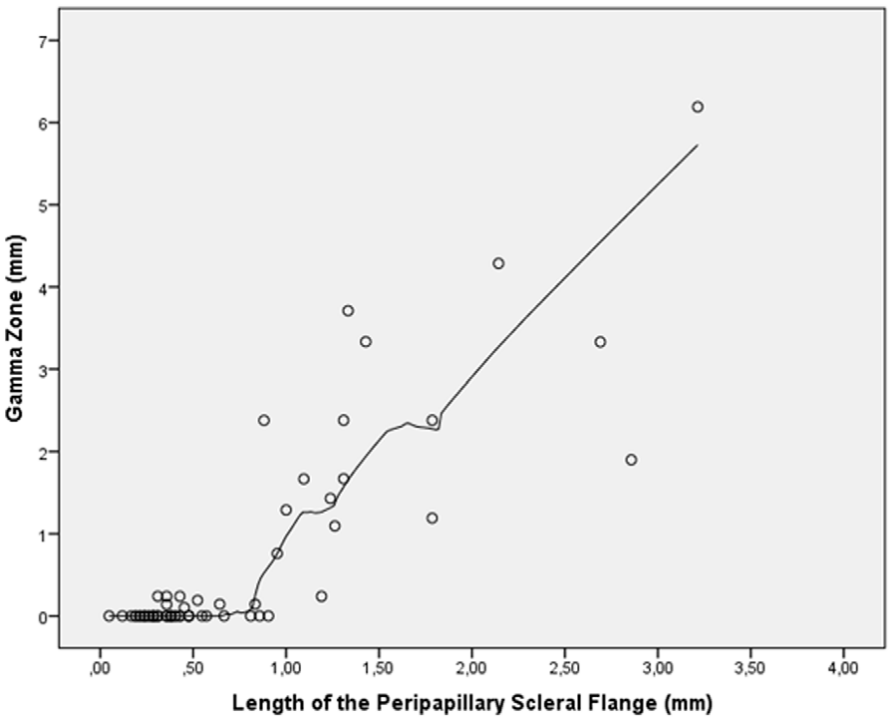
Scatterplot showing the distribution size of gamma zone versus length of the peripapillary scleral flange (locally weighted scatterplot smoothing (LOESS) statistics).

Mean length of gamma zone was 0.63±1.25 mm (median: 0; range: 0 to 6.19 mm). In univariate analysis, gamma zone was not significantly associated with age (*P* = 0.84) nor gender (*P* = 0.76). In the non-highly elongated group (axial length ≤26.0 mm), the glaucomatous group and the non-glaucomatous group did not vary significantly in size of gamma zone (0.03±0.08 mm versus 0.03±0.07 mm; *P* = 0.71). In a similar manner in the highly elongated group (axial length >26.0 mm), the glaucomatous group and the non-glaucomatous group did not vary significantly in size of gamma zone (0.75±1.21 mm versus 0.83±1.18 mm; *P* = 0.86). Gamma zone size was not significantly related with vertical globe diameter (*P* = 0.18) and horizontal globe diameter (*P* = 0.08). Size of gamma zone significantly increased with axial length in a non-linear manner (*P*<0.001; quadratic regression correlation coefficient r^2^ = 0.73). In a LOESS (locally weighted scatterplot smoothing) statistics, the increase in gamma zone size started at an axial length of about 26.5 mm (Fig. 5). Gamma zone size was significantly correlated with the thickness of the peripapillary scleral flange (*P*<0.001; quadratic regression correlation coefficient r^2^ = 0.68). In a LOESS statistics, the increase in gamma zone size started at a thickness of the peripapillary scleral flange of 0.3 mm or less (Fig. 6). Size of gamma zone significantly increased with the length of the peripapillary scleral flange (*P*<0.001; r^2^ = 0.74; equation of the regression line: Length of Gamma zone = 1.61×Length of Scleral Flange −0.52 mm). The diagram showed a steep increase in the length of gamma zone starting a scleral flange length of 0.8 mm (Fig. 7).

In univariate analysis, size of gamma zone was significantly associated with the thickness of the sclera measured at the posterior pole (*P*<0.001; correlation coefficient r = −0.73), outside of the optic nerve head after merging of the optic nerve sheaths with the sclera (*P*<0.001; r = −0.67), and at the midpoint between the posterior pole and the equator (*P*<0.001; r = −0.64). Gamma zone size was not significantly associated with the sclera thickness measured at the equator (*P* = 0.38), ora serrata (*P* = 0.25), and limbus (*P* = 0.95).

In multivariate analysis, gamma zone size remained to be significantly associated with axial length (regression coefficient B: 0.13 (95%CI: 0.06, 0.21); standardized coefficient beta: 0.45; *P*<0.001) and scleral thickness at the posterior pole (B: −1.95 (95%CI: −2.99, −0.91); beta: −0.46; *P*<0.001). Similar results were found for scleral thickness measurements obtained outside of the optic nerve head after merging of the optic nerve sheaths with the sclera (B: −1.26 (95%CI: −2.31, −0.08); beta: −0.30; *P* = 0.02), while the scleral thickness measured at the midpoint between the posterior pole and the equator was no longer significantly (*P* = 0.34) associated with the size of gamma zone after adjustment for axial length.

Delta zone was present only in eyes (n = 20) with an axial length of ≥27 mm and a peripapillary scleral flange length of more than 0.80 mm. Its mean length was 0.47±0.80 mm; median: 0.00; range: 0 to 3.21 mm). In univariate analysis, delta zone length was consequently significantly associated with axial length (*P*<0.001), thickness of the peripapillary scleral flange (*P* = 0.001), and with the thickness of the sclera at the posterior pole (*P*<0.001), outside of the optic nerve head after merging of the optic nerve sheaths with the sclera (*P*<0.001), and at the midpoint between the posterior pole and the equator (*P* = 0.002). Delta zone size was not significantly associated with age (*P* = 0.65), glaucoma (*P* = 0.15), sclera thickness measured at the equator (*P* = 0.48), ora serrata (*P* = 0.31), and limbus (*P* = 0.84). In multivariate analysis, delta zone size remained to be significantly associated with axial length (B: 0.03 (95%CI: 0.01, 0.05); beta: 0.14; *P* = 0.001) and scleral flange length (B: 1.04 (95%CI: 0.95, 1.14); beta: 0.87; *P*<0.001). If presence of glaucoma or age were added as independent parameter to this multivariate analysis model, neither presence of glaucoma (*P* = 0.73) nor age (*P* = 0.60) were significantly associated.

## Discussion

Our histological results on a parapapillary region (“gamma” zone) between the border of the optic nerve (as defined by the outer surface of the pia mater) and the beginning of Bruch's membrane confirms previous clinical studies and histologic single case descriptions [7]–[15]. Using spectral-domain optical coherence tomography, Hayashi and colleagues examined the peripapillary atrophy in 100 patients with primary open-angle glaucoma and in 100 normal subjects [13]. They found that parapapillary beta zone according to their definition was composed of straight or downward-curved Bruch's membrane in 68 eyes or of a downward-bending slope lacking Bruch's membrane in 79 eyes. This latter region without Bruch's membrane was termed “gamma” zone in our study. In Hayashi's study, presence of glaucoma and less myopic refractive error were associated with the curved-type of Bruch's membrane, and the region without Bruch's membrane (called gamma zone in our study) was associated with myopic refractive error. These clinical findings agree with our results in that gamma zone was strongly associated with axial length but not with glaucoma, while our beta zone was correlated with glaucoma, however not with elongated axial length. Park and colleagues assessed the microstructural anatomy of clinical beta zone parapapillary atrophy by using Fourier-domain optical coherence tomography [9]. They found that the edge of Bruch's membrane did not extend to the optic disc margin in all eyes, what would be the equivalent of gamma zone in our histological study. Lee and coworkers evaluated the cross-sectional configurations of peripapillary atrophy alpha zone and beta zone in normal subjects using spectral domain-optical coherence tomography [8]. Among other findings, they reported on slope and step configurations of the scleral bed and hump- and wedge-shaped appearances of Bruch's membrane in the peripapillary region, and that the presence of the step configuration was associated with myopia and longer axial length. This step configuration resembled gamma zone in our histological study.

Interestingly, gamma zone in our study was strongly associated with axial length with a steep increase starting at an axial length of 26.5 mm (Fig. 5). The cut-off value of an axial length of 26.5 mm is similar to the cut-off values of about −8 diopters for the differentiation between medium myopia and high myopia as suggested in clinical studies [16], [17].

Delta zone as described and defined in our histological study, has not been described in clinical studies using optical coherence tomography yet. The marked thinning and elongation of the peripapillary scleral flange in delta zone may be associated with the increased glaucoma susceptibility in highly myopic eyes as shown clinically [18], since the peripapillary scleral flange acts as the biomechanical anchor of the lamina cribrosa [19], [20]. Future studies with a larger sample size may address that topic. In addition to the thinning of the peripapillary scleral flange, the paucity of larger vessels in delta zone and the increased distance between the peripapillary arterial circle of Zinn-Haller and the optic disc border in highly myopic eyes (Fig. 4) may be an additional factor for the increased glaucoma susceptibility in highly myopic eyes [21].

Interestingly, beta zone as defined in our histological study was associated with glaucoma and it was not associated with myopia. It may suggest that the histological changes observed in histological beta zone, i.e., loss of RPE cells and photoreceptors and a closure of the choriocapillaris may be related to the glaucomatous optic neuropathy. Since clinical beta zone has up to now summarized the histological beta zone, gamma zone and delta zone, and since gamma zone and delta zone were not related with glaucoma in the present study, one may infer that a clinical differentiation between beta zone, gamma zone and delta zone may increase the clinical diagnostic value of a re-defined clinical beta zone (without gamma zone and delta zone) for the diagnosis of glaucoma. Interestingly, the region of Bruch's membrane with the underlying choriocapillaris occluded was significantly smaller than beta zone (defined as Bruch's membrane without RPE cells). It suggests that a complete loss of RPE cells may occur earlier than a complete closure of the choriocapillaris. It could indicate that a loss of RPE cells would lead to the closure of the choriocapillaris since an intact choriocapillaris depends on an intact RPE layer. One has to clearly keep in mind however, that this is a morphologic study and that disturbances in the blood perfusion of the choriocapillaris may have first led to a damage and loss of the RPE, which could then lead to the choriocapillaris closure.

Potential limitations of our study should be mentioned. First, due to postmortem swelling of the tissue after enucleation and due to the histological preparation of the slides, the measurements given in this study will not represent dimensions as determined intravitally. It was, however, not the purpose of our investigation to evaluate the measurements of the peripapillary structures and the sclera in real dimensions, but to compare the measurements of the various structures with each other. Second, the study did not include normal human eyes but eyes which were enucleated either due to a malignant choroidal melanoma or due to end-stage glaucoma. It is, therefore, not clear whether the results of our study can be generally transferred onto normal human eyes. Third, the measurements were not masked since the optic nerve and the retinal nerve fiber layer could not be covered when the measurements were taken. A bias could therefore have been introduced. Fourth, serial sections of the globes were not available so that it was not possible to determine, whether the histological section was located in the very center of the optic disc or whether it ran slightly paracentrally. Fourth, axial length was measured using calipers, so that the measurement included retinal, choroidal and scleral thickness at the posterior pole. This was different from the usual clinical measurement of axial length as linear distance from the anterior apex of the cornea to the anterior surface of the opposite retina. Also, the measurement was influenced by the fixation induced shrinkage of the globe.

In conclusion, parapapillary gamma zone (peripapillary sclera without overlying choroid, Bruch's membrane and deep retinal layers) was strongly related to axial globe elongation and was independent of glaucoma. Beta zone (Bruch's membrane devoid of RPE cells) was correlated with glaucoma and not with globe elongation. Since the region with occluded choriocapillaris was smaller than beta zone, loss of RPE cells may have occurred before complete choriocapillaris closure. Delta zone (no blood vessels >50 µm diameter) was present only in highly axially elongated globes. Future clinical studies may aim to differentiate ophthalmoscopically between the histological beta zone, gamma zone and delta zone, which all have been summarized into one clinical beta zone so far.
